# Further Account of the Case of Abstinence Contained in Our Last, with the Physiological and Pathological Remarks of Dr. Bourne

**Published:** 1808-12

**Authors:** 


### *Further Account of the Case of Abstinence contained in our last, with the Physiological and Pathological Remarks of* Dr. Bourne.

Ann Moor, a poor woman, aged 58, residing at Tutbury in the county of Stafford, by common report hath lived about eighteen months without taking any solid food whatever, and the greater part of the time also without liquids. In the early part of the history of her abstinence, she was accustomed to swallow occasionally small quantities of tea, and water; but this giving her considerable pain, she has for some months past washed her mouth only with a little cold water, two or three times a week, without swallowing any part of it. Such was the account I received a few weeks ago, and that the medical gentlemen in the town and neighbourhood, many having been to see her, were fully convinced of the truth of the story. Anxious to see so uncommon an instance of deviation from the natural laws of the human œconomy, I visited her on Monday the 17th instant, accompanied by Mr. Tylecote, Surgeon at Appleby, Leicestershire. From the vague and imperfect accounts we had each of us received of the situation of this poor woman, we expected to see an almost living skeleton, whereon were extended little more than the common teguments of the body. How great was our surprise then to behold a good-looking countenance; I do not mean to say a healthy one; and less general emaciation than may every day be witnessed in cases of fever, and in the last stage of a consumption. We had been informed, that upon laying the hand on the belly no traces of the bowels could be felt; but but that the integuments almost only remaining, the vertebræ of the spine, and the great artery descending along its course were palpable on the slightest pressure; insomuch that among the common reports, it is said that the viscera are drawn up into the chest. We found the state of these parts very different from the above description of them, as will appear by the subjoined account of our examination. And I suppose the difference in this respect from that given by some of her other medical examinators, arises from the occasional greater or less distension of the bowels themselves, and the tenseness or flaccidity of the skin over them. Her pulse beat 96 in the minute; was regular, rather small but hard, and underwent little or no variation during our examination. Her voice clear and strong, and respiration easy and free. Tongue somewhat dry and white in the middle, yet no thirst. She has frequently severe pain under the short ribs of the left side; increased on pressure there, and also by lying on the right side. She has sometimes head-ach, but when free from all pain can sleep very comfortably. Perspires a little occasionally on the hands and feet alone; and has sometimes general increased heat of the body, and flushings of the face. Her skin was then rather dry, as is found in a slight attack of fever. Placing our hands on the belly, the skin there was soft and loose; and the bowels plainly to be felt distended with air and some fluid, which evinced themselves also to our organs of hearing by a gurgling noise, similar to what happens by pressing on a membraneous tube containing air and water. And so far were the spine and great artery from being easily felt, it was only by considerable pressure that this could be done; which gave her great pain and nearly brought on fainting; and was followed by several eructations of flatulence; and she says, she sometimes has discharges of air per anum.

The following is the history of the case, as related by herself.

Some time prior to the summer of 1806, she was troubled with occasional pains of her sides and stomach, especially after eating, but her food was not then rejected; and she did not consider herself so ill as to pay any particular regard to these circumstances. She had had, for a long time, the care of a boy who had the evil, on whose body were a number of very offensive sores. At the above period her appetite began to decline, and what little food she took, her stomach nauseated, but did not reject; and she fancied it had the smell and taste of the ulcers of the scrophu-

[illegible]ous boy. In November following she felt herself unable to do her usual work, which was that of picking or beating cotton. In the month of March 1807, she was seized with fits, which by her description appear to have been epileptic; these continued about a fortnight, and were succeeded by cramps at the stomach and vomitings. She has had no fits since. About Easter of the same year, finding a total want of appetite, and much pain after swallowing, she gave up the attempt; and has never taken any thing solid since; nor has she had any desire for food. For some time she continued to take now and then a little tea and water, as mentioned above; but having now no thirst at any time, she contents herself with washing her mouth only two or three times a week. She has had no stool since the third of August, 1807; but she passes from half a pint to a pint of pale urine once in two or three days.

This extraordinary mode of life, as might be naturally expected, did not obtain general belief; and she was accordingly looked upon by many of her neighbours as an impostor. To dissipate this notion she consented to be removed to another house; where she was attended day and night for nearly three weeks, by persons consisting partly of medical men, and partly of such of her neighbours who disbelieved her story. These relieved each other regularly every four hours, and were satisfied that during the above period she took neither food nor drink, excepting once, by desire, she swallowed a spoonful or two of water; which gave her much pain.

Such are the circumstances attending this case, as collected by the testimonies above described; but, forming perhaps an unique instance of such protracted abstinence, and so very contrary to the established habits and indispensable wants of animated nature, many persons are yet nevertheless disposed to disbelieve the fact. If however there be any deception in the matter, it appears to be the most complete imposture ever practised. I think we can scarcely withhold our credit to the three weeks trial; and if so, I see no reason for doubting the truth of the whole. It is true indeed that, in many cases of disease, in fever especially, we find an almost total cessation of appetite for solids for three or more weeks; but then there is usually an increased desire for liquids; and at the termination of the disease, the general appetite returns. It is owing to a want of appetite entirely, I imagine, that the continuance of life here, under the privation of food, is to to be accounted for. For hunger, if not satisfied, is itself a stimulus, which would in time destroy the body; hence those unfortunate persons, who are completely deprived of the means of gratifying this appetite, quickly die. How then has life been sustained in the case before us? If we examine the subject philosophically, I think we may come to some rational conclusion. The elementary principles of the human body, or those into which it may be reduced, by means of chemistry, are very few. The food, whether solid or liquid, destined for its nutrition, and repair of the changes and waste it undergoes during life, is also resolvable into the same elementary principles. And these principles are moreover present in the atmosphere which we breathe, combined as it always is with watery vapour, &c. And it is only by the different combinations and modifications of these few elementary principles that the various articles of food; nay, the almost infinite variety in the products of nature, present themselves to our view; that one thing is sweet, one is sour, and another is bitter; this is soft and that is hard; one proves salutary, and another poisonous, &c. And according to the relative affinities which these elements have with each other, one is more readily than another acted upon by the juices of the stomach, intestines, &c.; or in familiar words, one is of more easy digestion than another. The universal receptacle for food, in mankind at least, is the stomach; but if the body be supplied through this medium with nutriment, containing only those principles which exist in the atmosphere which surrounds us; and if by the total want of appetite, this supply be precluded by the usual means; it is reasonable, or it is not unphilosophical at least, to suppose that life may be sustained, if the same principles can gain admission by other channels. It appears that the vigour and strength of the body cannot be maintained in this manner, as is the case with the subject under consideration; yet life itself may thus be preserved for an indefinite period. The well-known instances of hybernating animals afford proofs of its continuance for months by respiration alone.

I shall conclude with a few observations on two or three of the particulars noted in this case. In the first place, the nutrition of the system is evidently introduced by the lungs; it is not our purpose to inquire here by what means it is afterwards assimilated; she lives *apparently* on air alone; to use her own expression "she loves air," and has the chamber window constantly open. As she almost continually lies in bed, it is not probable that much is furnished ed by absorption from the general surface of the body. But it seems that a kind of digestive process is carried on in the intestinal canal; and that a species of chyle is there formed. That there is some secretion there, which undergoes a decomposition of its elementary principles, is apparent from the presence of air and moisture, which we discovered by pressing the abdomen with the hand; hence arise the frequent eructations of flatus, &c.; but from the tenuity of the matter imbibed, there are no gross particles to form what is usually evacuated by stool. The hardness of pulse and dry state of the skin, seem to indicate that there is not a sufficiency of moisture introduced into the system to answer the general intentions of nature; and probably owing to this defect, life will be gradually exhausted. I apprehend that a thickening, perhaps an ossification of the arterial system, has thus commenced, and will keep increasing; having begun at the most remote points from the heart, which afford the greatest resistance to the action of that organ; thence ascending to the greater vessels and to the heart itself; and from the gradual obliteration of these, life will become extinct in like manner to the burning out of a lamp. How long a time may be necessary for the completion of this process it is impossible to say; but from present appearances there is reason to believe she may continue some months longer. It is probable the frequent use of the warm bath, if she could bear it, would tend to defer her dissolution.

*Atherstone, Warwickshire, October* 27, 1808.

